# On failing in pregnancy

**DOI:** 10.1002/pmf2.70194

**Published:** 2025-12-05

**Authors:** Sarah N. Cross

**Affiliations:** ^1^ Department of Obstetrics Gynecology & Reproductive Sciences Division of Maternal Fetal Medicine Yale School of Medicine New Haven Connecticut USA

A colleague on Labor and Delivery recently sought my advice about one of her pregnant patients whom I was co‐managing. In giving me the update, she mentioned that the patient “failed her non‐stress test.” I was not surprised as I often hear this term on rounds, but it was the first time I'd heard someone refer to antepartum testing this way. The most common clinical scenario where I encounter this term is in relation to the diagnosis of gestational diabetes. Patients with gestational diabetes are frequently described as having failed the glucose tolerance test. Should they have tried harder? What does this term even mean in the broader medical context? Can one fail a medical evaluation as one does a school examination? How does this term make patients feel?

Other areas of Obstetrics and Gynecology where the word failure is used include trial of labor after cesarean (TOLAC), induction of labor (IOL), and even labor itself, as well as cerclage, noninvasive prenatal testing, in vitro fertilization (IVF) cycles, pessary, vaginal slings, and chemotherapy. A friend with ovarian cancer had been told she was failing chemotherapy. She felt defeated as though she was failing at her own healing. Unfortunately, she did not survive, but not because she failed.

The use of this term in medicine is not limited to Obstetrics and Gynecology. Physicians from a myriad of other fields have shared examples of how failure/failed is used when describing their patients. Babies are said to have failed the car seat test or the hearing screen. Even children with cancer can be described as failing induction or chemotherapy. In the emergency department patients are described as failing noninvasive ventilation and requiring intubation or failing outpatient treatment requiring admission. In the ICU patients can fail a swallow evaluation or have a failed breathing trial requiring prolonged intubation. Patients with treatment‐resistant depression might be described as having failed two or more antidepressants. To get approval from insurance companies for specialized medications or higher levels of care, we may have to document that our patients have failed initial therapies. The examples are endless.

This terminology is in problem lists and patient notes. MyChart® messages are sent to patients explaining that they have failed the glucose tolerance test. I have had friends and family recount these failures to me. And of course, my patients use this word to describe their results and themselves.

I understand the fear of failure. I have been fortunate to have had three pregnancies and have taken the glucose challenge three times. Each time I was deeply hopeful for a good result. This was partly because I know the work involved in having diabetes during pregnancy. But it was also partly because of what I thought it would mean about me, about failing.

According to the Oxford English Dictionary fail (verb) means “to be inadequate or insufficient; to disappoint; to be wanting or deficient in; to be unsuccessful” [1]. While our training might frame the medical evaluation as tests to be passed or failed, many patients think of themselves as the failure.

When an estimated one‐third of pregnant people suffer from anxiety [2], it is just a small psychological step to internalize these failures. One of the questions on the commonly used Edinburgh Postnatal Depression Scale (EPDS) is “I have blamed myself unnecessarily when things went wrong.” [3] In a larger context, our society has a history of blaming parents, mothers, for the challenges, and even medical problems, their children face. For example, while the 1940s theory that a mother's emotional coldness or frigidity was the cause of autism—the so‐called refrigerator mother theory—has been debunked, now our patients worry about acetaminophen. When lactation is especially challenging, we might feel responsible for our own precious newborns' failure to thrive.

In medicine our conversations with patients are at least as important as our medical knowledge and clinical skills. The words we choose in our counseling and in our charts carry great weight in our patients’ hearts and our colleagues’ minds. This is all the more true since the passage of the 21st Century Cures Act whereby our patients can read every word we use. What we say and now what we document must be carefully weighed. The language we use has power; it contributes to patient narratives—what they hold true about themselves. Language is a tool, and also an opportunity for us to promote health equity. Both the CDC's Health Equity Guiding Principles for Inclusive Communication [4] and the AMA's Advancing Health Equity: A Guide to Language, Narrative and Concepts [5] encourage us to avoid unintentional blaming. Practicing trauma‐informed care shifts the focus from what is wrong with the patient to what has happened to the individual [6].

If we do not want our patients to blame themselves, to feel like a failure, why do we use the word failure? Instead of failure, when discussing gestational diabetes, we can describe results as elevated; other options include above range or high. Medications or treatments may be attempted, unsuccessful, ineffective, or nontherapeutic. For example, IOL, IVF, and cerclage could be attempted or unsuccessful. Similarly, a TOLAC can be attempted or unsuccessful. Laboratory, imaging, and other medical tests could be called laboratory and imaging evaluations or assessments. Similarly, antepartum testing could be antepartum assessment. When describing labor, there has already been some change; instead of failure to progress more often we say arrest of dilation or descent.

Indeed, language is not static, it evolves over time, both across generations, but also within a person's lifetime [7]. Although much language acquisition occurs in childhood, adults continue to learn a surprising number of new words [8] and the meanings of these words change, partially drive by social context [9, 10]. The very act of us talking to each other is part of how language changes [11].

Medical students, specifically, learn a staggering number of new words, by some estimates more than those studying a foreign language [12]. In addition to this new vocabulary, they are also learning patient‐centered communication. While each of us adjusts our vocabulary to ensure that it is contemporaneous and patient‐centered medical trainees are a perfect group to first learn and then help spread these changes in terminology as they become our colleagues.

In pursuit of patient safety, to minimize future harm, we do need to lean into failure in health care. Evaluating adverse outcomes allows us to avoid preventable injuries in the future. A significant proportion of severe maternal morbidity is due to health care failures [13]. It might be appropriate to describe the shortcomings of our complicated, imperfect health care system as failures. Even outcomes that are not serious safety events, such as unsuccessful treatments, might be failures. Perhaps my friend died of ovarian cancer because the chemotherapy failed her. However, while our patients may suffer the effects of medicine's errors, its inadequacies, its limitations, its failures, they do not fail.

## CONFLICT OF INTEREST STATEMENT

The author declares no conflicts of interest.

## FUNDING INFORMATION

The author received no specific funding for this work.

## ETHICS STATEMENT

Institutional approval was not sought for this work as it is a viewpoint/opinion and not research. This work is entirely that of the author.

## Data Availability

Data sharing is not applicable to this article as no datasets were generated or analyzed during the current study.
